# The role of adjuvant chemotherapy in small bowel adenocarcinomas

**DOI:** 10.1093/oncolo/oyag262

**Published:** 2026-07-09

**Authors:** Kruti Vora, Ryan H Moy, Alfred I Neugut

**Affiliations:** Department of Medicine and Herbert Irving Comprehensive Cancer Center, Vagelos College of Physicians and Surgeons, Columbia University, New York, NY 10032, United States; Department of Medicine and Herbert Irving Comprehensive Cancer Center, Vagelos College of Physicians and Surgeons, Columbia University, New York, NY 10032, United States; Department of Medicine and Herbert Irving Comprehensive Cancer Center, Vagelos College of Physicians and Surgeons, Columbia University, New York, NY 10032, United States; Department of Epidemiology, Mailman School of Public Health, Columbia University, New York, NY 10032, United States

The small bowel is situated anatomically between two organs that have among the highest incidence rates of cancer in the world—the stomach and the large bowel. And yet the incidence rates of cancer in this organ, which by area is the largest organ in the gastrointestinal (GI) tract, are extraordinarily low. Why is this? Several hypotheses have been suggested: the small bowel is relatively sterile in contrast to the stomach or large bowel; there is rapid transit time of bowel contents through the small intestine, reducing exposure to the carcinogens that it contains; the small intestine has a large amount of immune tissue, perhaps suppressing early malignant transformation; and small bowel enterocytes have high turnover rates with robust apoptosis mechanisms.^1–3^

Small bowel cancers ultimately make up only 0.6% of all cancers in the United States, with 30%-40% of these being adenocarcinomas.^4^ Development of duodenal adenocarcinoma is an even rarer phenomenon, making up about half of small bowel adenocarcinomas.^5–6^ Unfortunately, patients with duodenal adenocarcinoma have worse overall survival compared to jejunal and ileal tumors; it is unclear if this is due to innate tumor biology, increased prevalence in the elderly, or more complex surgical resection.^7^

Because of its rarity, relatively few treatment trials have been conducted for small bowel adenocarcinomas, so the current standard of care is modeled off the treatment of large bowel adenocarcinomas. Adenocarcinoma of the small bowel resembles adenocarcinoma of the large bowel in several ways: both tumors can arise from the adenoma-carcinoma sequence, both are elevated in the polyposis genetic syndromes and inflammatory bowel disease, and both have overlapping genetic mutations (KRAS, TP53, SMAD4).^8^ There are also some key differences between large and small bowel tumors. For example, small bowel adenocarcinomas have lower rates of APC/WNT pathway mutations and have higher rates of microsatellite instability-high (MSI-H) disease, pointing toward alternative tumorigenesis mechanisms.^8^

Nevertheless, the current standard of care for small bowel adenocarcinoma resembles large bowel cancer treatment. We opt for no chemotherapy for stage I disease; occasional adjuvant therapy for stage II; a consistent recommendation of adjuvant chemotherapy for those with stage III disease; and palliative chemotherapy for those who are stage IV or recurrent. For MSI-H metastatic small bowel cancers, we consider the use of immunotherapy.

In this issue of *The Oncologist*, Gu and colleagues present an analysis of duodenal adenocarcinoma cases treated in China and propose a predictive model for short- and long-term outcomes for patients who have undergone surgical resection.^9^ Their nomogram stratifies patients into low- and high-risk groups based on several variables: age, intraoperative blood transfusion, operative time, AST on post-operative day (POD) 1, post-operative hemorrhage, perineural invasion, microvascular invasion, and tumor recurrence. This study offers a valuable prognostic tool for patients with small bowel cancer undergoing surgery in China. However, the study does not include in its modeling an analysis of adjuvant chemotherapy. As GI oncologists, after review of this paper, we want to ensure that readers of this journal walk away with an appreciation of the contribution that systemic chemotherapy may make in improving outcomes for patients with small bowel adenocarcinoma.

Prospective studies investigating adjuvant therapy for resected duodenal adenocarcinoma are ongoing, though accrual remains difficult given the rarity of this tumor. The GLOBAL BALLAD trial is an international phase III randomized controlled trial (RCT) that launched in 2015 to compare observation, fluoropyrimidine monotherapy, and FOLFOX in resected stage I–III small bowel adenocarcinoma. However, the trial closed early due to poor accrual, and the results from this study have not yet been reported. In the absence of prospective data, treatment decisions in the adjuvant setting rely on retrospective analyses and extrapolation from large bowel studies.

One large retrospective study used the National Cancer Database (NCDB) to study 4746 patients with resected stage I–III small bowel adenocarcinoma, 1674 of whom received adjuvant chemotherapy rather than surgery alone. Across the entire cohort, there was an overall survival benefit with the addition of adjuvant chemotherapy versus surgery alone (median overall survival [mOS] 63.2 vs. 44.5 months, *P* < .001), largely driven by patients with stage III disease (mOS 42.4 vs. 26.1 months, *P* < .001).^7^ While there was no statistically significant survival difference for patients with stage I or stage II disease, the outcomes numerically favored adjuvant chemotherapy. In subgroup analyses of patients with duodenal adenocarcinoma, the overall survival benefit persisted among those with stage III disease (34.1 vs. 24.3 months, *P* = .002).

Another small retrospective study at Duke University Medical Center analyzed 32 patients with duodenal adenocarcinoma, 16 of whom received surgery alone while 16 received either pre- or post-operative 5-fluorouracil (5-FU) based chemotherapy and radiation therapy. While sample sizes were small, the patients undergoing R0 resection had overall survival benefit from perioperative chemotherapy and radiation therapy (5-year OS 83% vs. 53%, *P* = .07).^10^

Other studies, however, showed less benefit with adjuvant chemotherapy. For example, one retrospective study by Zaanan and colleagues showed that only patients with high-risk stage II and stage III jejunal and ileal adenocarcinomas benefit from adjuvant therapy, but not those with duodenal tumors.^11^ Two other studies also showed no survival benefit with adjuvant chemotherapy across small bowel adenocarcinomas.^5^^,^^12^

Based off the current available retrospective data and extrapolation from the current standard of care for large bowel cancers, the National Comprehensive Cancer Network (NCCN) guidelines recommend 3-6 months of adjuvant chemotherapy for all patients with node-positive duodenal adenocarcinoma or tumors with high-risk features: positive margins, <5 lymph nodes examined, bowel perforation, lymphovascular invasion, perineural invasion, or poorly differentiated histology. The chemotherapy agent of choice is a fluoropyrimidine-based regimen. Fluoropyrimidines inhibit thymidylate synthase, which is broadly expressed in glandular epithelial tumors, and have shown notable activity against large bowel adenocarcinomas. Phase II trials have also demonstrated responses to fluoropyrimidine and platinum-based chemotherapy in patients with metastatic small bowel adenocarcinoma.^13–15^

There is active investigation into the use of other chemotherapy agents as well; this issue of *The Oncologist* includes a study from investigators at MD Anderson Cancer Center evaluating the potential utility of taxanes for treatment of small bowel adenocarcinomas.^16^ Taxanes have not been a major player in the management of large bowel cancer, in part due to resistance mechanisms such as loss-of-function APC gene mutations.^17^ Small bowel cancers, however, have lower APC mutation rates and are thus hypothesized to be more taxane-sensitive. One phase II study observed partial responses with nab-paclitaxel therapy in two of 10 patients with small bowel adenocarcinoma.^18^ In this issue, Grandhi, Lim, and colleagues retrospectively evaluated 70 patients with small bowel adenocarcinoma treated with taxane therapy at MD Anderson Cancer Center. The study demonstrated a 24% overall response rate, with a median time to progression of 3.1 months and a median overall survival of 8.7 months. Although response rates remain modest, this study is an important contribution to the field and supports further investigation of taxanes as a later-line or salvage therapy option for small bowel adenocarcinoma.

Duodenal adenocarcinoma remains a rare and understudied malignancy for which high-quality prospective evidence is lacking. The prognostic nomogram presented by Gu and colleagues is a meaningful step forward in the risk-stratification of patients after surgical resection; however, clinicians should view it as one component of a broader treatment strategy. Retrospective data support the use of adjuvant fluoropyrimidine-based chemotherapy in node-positive and high-risk resected disease, and there is emerging data for use of other chemotherapeutic agents as well. Ultimately, patients with duodenal adenocarcinoma are best served by a multidisciplinary approach and by the continued prioritization of dedicated prospective trials in this understudied disease.
